# High response rate and low cost of WeChat-based data collection of infant and young child feeding data in rural Qinghai Province, China

**DOI:** 10.7189/jogh.12.11011

**Published:** 2022-10-21

**Authors:** Jian Zhang, Qiong Wu, Xiaotong Wang, Yanfeng Zhang, Michelle Helena van Velthoven

**Affiliations:** 1 Department of Integrated Early Childhood Development, Capital Institute of Pediatrics, Beijing, China; 2 Nuffield Department of Primary Care Health Sciences, University of Oxford, Oxford, United Kingdom

## Abstract

**Background:**

Measuring the infant and young child feeding (IYCF) indicators is key to effectively tracking the progress of child health programs and making evidence-based decisions. WeChat is the most popular mobile social media platform in China and has become a promising tool for collecting health data. This study aims to explore the response rate and costs of a WeChat-based questionnaire on IYCF information in rural China.

**Methods:**

We conducted two cross-sectional surveys with caregivers of children aged 6-23 months in two rural counties in Qinghai Province (Menyuan and Datong County), China, from January to March 2022. Both surveys used the same WeChat IYCF questionnaire to collect household information, infant feeding practices, and caregivers’ feeding knowledge. Village doctors sent a quick response (QR) code to caregivers that was linked to the WeChat IYCF questionnaire. Participating caregivers scanned the QR code using their own WeChat account on their smartphone and filled in the questionnaire online. If they could not use WeChat themselves, village doctors administered the questionnaire. Once caregivers finished the questionnaires, they received a personalized report with infant feeding recommendations.

**Results:**

We recruited 1274 caregivers of children aged 6-23 months in Menyuan County and 1748 caregivers in Datong County. The total response rate in the two counties was 98.2%; 77.6% of questionnaires were self-administered by caregivers and 20.6% were interviewer-administered by village doctors. The questionnaires were filled in twice by 209 (6.9%) caregivers. The cost of these two WeChat IYCF surveys was much lower than the cost of a previously conducted face-to-face survey: 11.8 yuan (US$1.85) in Menyuan County and 7.5 yuan (US$1.18) in Datong County for the WeChat survey vs 112.7 yuan (US$17.70) for the face-to-face survey in Huzhu County.

**Conclusions:**

This study showed that using WeChat for IYCF surveys can achieve a very high response rate at a low cost in rural China. Village doctors played a very important role in achieving this high response rate. Providing feedback to caregivers may improve their feeding practices and this intervention could be incorporated into the data collection process.

Infant and young child feeding (IYCF) is crucial to the health of children in their early stages of life, and inappropriate feeding can lead to health problems such as malnutrition, growth retardation, obesity, infection, and iron deficiency anaemia [1]. In 2003, the World Health Organization (WHO) and the United Nations Children's Fund (UNICEF) jointly issued the Global Strategy for Infant and Young Child Feeding. This strategy recommends exclusive breastfeeding for six months after birth, adequate and safe complementary food starting from the age of six months, and continued breastfeeding for children aged two months or above [2]. To measure the coverage of these feeding recommendations, a set of standard IYCF indicators and the guidelines for data collection and analysis were published in 2010 and updated in 2021 by WHO and UNICEF [3,4]. Measuring the IYCF indicators is key to effectively tracking the progress of child health programs and making evidence-based decisions [5]. Recent data on these indicators showed that the exclusive breastfeeding rate of infants younger than six months was 41% worldwide [6], 37% in low- and middle-income countries (LMIC) [7], and 29.2% in China [8]. The proportion of children aged 12-23 months who received continued breastfeeding was 68.3% worldwide [9]. However, the proportion of children who were breastfed at 1 year of age was only 11.5% and 6.9% at 2 years of age in China [10]. For complementary feeding, the proportion of infants aged 6-8 months who receive solid or semi-solid foods was 64.5% worldwide [9], and 83.8% in poor rural regions in China [11]. The proportions of children who met minimum dietary diversity (MDD), minimum meal frequency (MMF), and minimum acceptable diet (MAD) were 28.2%, 50.3%, and 15.9% worldwide in 2017, respectively [4,9]. The proportion in China were 34.5% for MDD, 69.2% for MMF and 23.7% for MAD [12].

Traditionally, data on these indicators are collected by conducting interviewer-administered face-to-face household surveys, which are labour-intensive, time-consuming, and costly [13]. With the rapid development of the Internet and new media, electronic online survey questionnaires are increasingly available and are gradually replacing traditional face-to-face surveys [14]. As the most popular mobile social media platform in China, WeChat has become a promising tool for collecting electronic data [15,16]. WeChat has over 1.2 billion monthly active users, and more than 60% of WeChat users open the app more than 10 times a day [17]. The widespread use of WeChat supports its potential use for administering questionnaires [18], especially for individuals who do not have computer experience and skills [16]. In 2021, our research team developed a WeChat self-administered IYCF questionnaire based on the WHO/UNICEF standard questionnaires and conducted a small-scale test-retest study. That study demonstrated that most questions in the WeChat IYCF questionnaire and the calculated key IYCF indicators showed very good agreement between the interviewer-administered survey and the WeChat self-administered survey [19]. However, the response rates of the WeChat IYCF questionnaire in large-scale population surveys are unknown. Response rates are fundamental in determining the validity of the findings from questionnaire surveys [20,21] and achieving a high response rate is important when conducting surveys [22].

## METHODS

### Study design

We aimed to assess the response rates and costs of WeChat self-administered questionnaire surveys on IYCF information. This study was carried out between January 12, 2022, and March 21, 2022, in Qinghai Province, China. We conducted two cross-sectional population-based questionnaire surveys with caregivers of children aged 6-23 months in Menyuan County and Datong County. Both surveys used the same WeChat IYCF questionnaire to collect information on households, infant feeding practices, and caregivers’ feeding knowledge.

### Study setting

Qinghai Province is located in Northwest China, with a total population of 5.93 million in 2020. It has 34 counties and 439 townships. The Qinghai resident per capita disposable income in 2020 was ¥12 342 (US$1939.19,) for rural people [23], far lower than the average national income (¥17 131; US$2691.65) [24].

Menyuan County is located in the northeast of Qinghai Province and has 12 townships and 105 villages. In 2020, the total population was 138 335, 62.3% (86 117) of which belonged to the rural population [25]. There were 1493 children aged 6-23 months in rural Menyuan County (unpublished data).

Datong County is located in the east of Qinghai Province and has 20 townships and 289 villages. In 2020, the total population was 403 368, 49.0% (197 639) of which belonged to the rural population [26]. There were 6375 children aged 6-23 months in rural Menyuan County (unpublished data).

### Participants

We invited caregivers of children aged 6-23 months old in Menyuan County and Datong County to participate in this study. We excluded caregivers who: 1) moved out of the county; 2) lived in urban areas; 3) had children aged younger than six or older than 23 months.

### Sample size and sampling

The sample size for the survey was based on the key IYCF indicator of MDD, which was 53.7% in 2013 according to the China National Nutrition and Health Survey [10]. We used a desired level of absolute precision of 0.04, with a power of 80% and a significance level of 5%. We calculated that a sample size of 1212 children aged 6-23 months would be sufficient in each county. Furthermore, we oversampled 30% to compensate for possible refusal and loss to follow-up.

In Datong County, we selected 87 villages from 289 villages using proportional to population size sampling, and surveyed all the caregivers of children aged 6-23 months in those villages. In Menyuan County, there were fewer children aged 6-23 months old; therefore, we recruited all eligible caregivers who met our inclusion criteria.

### Recruitment

Before recruitment, we obtained an original list of names of all children aged 6-23 months in rural areas in both counties based on the local routine information system, which included information on county, township, village, child’s name, date of birth, gender, and mothers’ telephone number. We asked village doctors to cross-check the name list and add information on main caregivers and whether the village doctor added the main caregiver on WeChat as a “friend” contact. Village doctors returned the final name list. A total of 1493 children were listed in the 105 villages in Menyuan County and 1868 in 87 villages in Datong County.

Based on the inclusion and exclusion criteria, we excluded 219 children from the list in Menyuan County (50 moved out of the county, 31 were younger than six months, and 138 were older than 23 months) and excluded 120 children in Datong County (11 moved out of the county, 75 were younger than six months, and 34 were older than 23 months). Finally, 1274 children aged 6-23 months in Menayuan County and 1748 children in Datong County were eligible for the study.

### WeChat IYCF questionnaire

The WeChat IYCF questionnaire used in this study was based on the adapted WHO Maternal, Newborn and Child Health Household Survey (MNCHHS) (unpublished, 2009) and the Indicators for assessing infant and young child feeding practices (WHO&UNICEF) [3]. We compared the agreement of this WeChat questionnaire with the interview-administered survey in our previous study, which showed that six key IYCF indicators had a substantial or almost perfect agreement (κ = 0.78-0.94): MDD, MMF, MAD, consumption of iron-rich or iron-fortified foods, continued breastfeeding at one year, and continued breastfeeding at two years [19].

The questionnaire started with asking for informed consent, followed by 58 questions, including 21 questions about basic information, 36 questions on breastfeeding and complementary feeding knowledge, practices, and information sources, and one question about the respondent.

We set the questionnaire up on the online survey tool Sojump (http://www.sojump.com) platform. We obtained a quick response (QR) code that was used as a link to the questionnaire. Participants could scan the QR code using their WeChat app and then fill in the questionnaire online. When they finished and submitted the questionnaire, they could get a personalized feeding assessment report based on their answers, which consisted of scores for each question, a total score for all questions, and personal feeding problems and recommendations.

### Data collection

**Figure 1:**
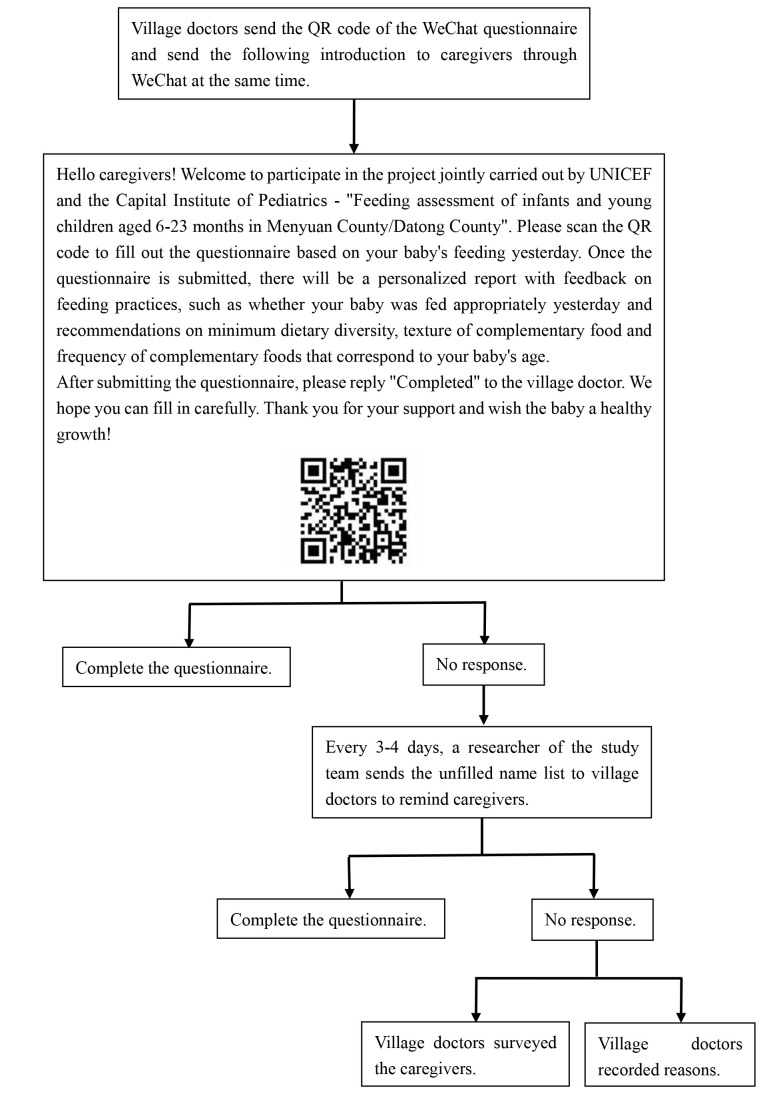
The flowchart of distribution of the WeChat questionnaire.

We conducted the surveys in Menyuan County between January 12 and March 1, 2022, and in Datong County between February 28 and March 21, 2022. We distributed the WeChat IYCF questionnaire through the three-tier health care system (county-township-village). We made a flowchart for distributing the WeChat questionnaire (**Figure 1**) and sent it to the county coordinators at the County Maternal and Child Health hospitals. Then the county coordinators sent the flowchart to the township coordinators who forwarded it to the village doctors. In the flowchart, village doctors were asked to send the WeChat questionnaire QR code to caregivers and the introduction of the survey, informing caregivers that they would receive a personalized report with feedback on feeding practices after finishing the questionnaire. Based on the name list, village doctors sent the introduction and the QR code to all the eligible caregivers in their villages by WeChat. Participating caregivers scanned the QR code using their own WeChat and filled in the questionnaire online.

Once the participants submitted the questionnaire, the research team could immediately see the data on the Sojump platform. Every three to four days, one research member of study team downloaded the data on the platform and checked who had finished the surveys. Using the original name list, the research member developed a new name list of caregivers who did not fill in the questionnaire and sent this back to village doctors; they then reminded those caregivers to fill in the questionnaire by sending the introduction and the QR code again. For caregivers who could not use WeChat or were illiterate, village doctors surveyed them face-to-face by using the WeChat questionnaire. At the end of the study, we asked the village doctors to record the reasons for not completing the questionnaire for caregivers who did not fill in the questionnaire after five reminders.

### Outcomes

The main outcome of this study was the response rate of the WeChat IYCF questionnaire, defined as the proportion of participants who completed the questionnaire, either by caregivers themselves or with the help of village doctors.

The secondary outcome was the cost of the WeChat IYCF surveys in both counties, which included payment of one supervisor, two county coordinators, 32 township coordinators, and 192 village doctors, and a subscription to the Sojump online survey tool. We calculated the cost per WeChat IYCF questionnaire in each county by dividing the survey’s total cost by the number of respondents.

We also compared the costs, labour, and time spent between the WeChat IYCF surveys and a similar face-to-face survey that we previously conducted in Huzhu County, Qinghai, in 2018. This 2018 survey included 754 caregivers of children aged 6-23 months from 38 villages, and it took one training day and six survey days [27]. The cost of 2018 survey included labor payment (two supervisors, 26 interviewers, one province coordinator, two county coordinators, 36 township coordinators, and 38 village doctors), transportation fees (from Beijing to Huzhu County, from Xining to Huzhu County, and from county to villages), hotel fees for the supervisor and interviewers, meal fees, insurance fees, gift fees for caregivers, and the survey software subscription. We compared the total cost and cost per questionnaire of the two survey methods.

The third outcome was the six key IYCF indicators, which were updated by the WHO and UNICEF in April 2021 (**Box 1**).

Box 1 **WHO key IYCF indicators in 2021**

1. Introduction of solid, semi-solid, or soft foods (ISSSF) 6-8 months: percentage of infants 6-8 months of age who consumed solid, semi-solid, or soft foods during the previous day.

2. Minimum dietary diversity (MDD) 6-23 months: percentage of children 6-23 months of age who consumed foods and beverages from at least five out of eight defined food groups during the previous day. The eight food groups used for tabulation of this indicator are: 1) breast milk; 2) grains, roots, tubers, and plantains; 3) pulses (beans, peas, lentils), nuts, and seeds; 4) dairy products (milk, infant formula, yogurt, cheese); 5) flesh foods (meat, fish, poultry, organ meats); 6) eggs; 7) vitamin-A rich fruits and vegetables; and 8) other fruits and vegetables.

3. Minimum meal frequency (MMF) 6-23 months: percentage of children 6-23 months of age who consumed solid, semi-solid, or soft foods (but also including milk feeds for non-breastfed children) at least the minimum number of times during the previous day. The minimum number of times is defined as: 1) two feedings of solid, semi-solid, or soft foods for breastfed infants aged 6-8 months; 2) three feedings of solid, semi-solid, or soft foods for breastfed children aged 9-23 months; 3) four feedings of solid, semi-solid, or soft foods or milk feeds for non-breastfed children aged 6-23 months, whereby at least one of the four foods must be a solid, semi-solid, or soft food.

4. Minimum acceptable diet (MAD) 6-23 months: percentage of children 6-23 months of age who consumed a MAD during the previous day. The MAD is defined as: 1) for breastfed children: receiving at least the MDD and MMF for their age during the previous day; 2) for non-breastfed children: receiving at least the MDD and MMF for their age during the previous day as well as at least two milk feeds.

5. Consumption of iron-rich or iron-fortified foods (CIRIFF): percentage of children aged 6–23 months who received iron-rich food or iron fortified food that was specially designed for infants and young children, or that was fortified in the home.

6. Continued breastfeeding (CBF) 12-23 months: percentage of children 12-23 months of age who were fed breast milk during the previous day.

### Statistical analysis

Questionnaire data uploaded to the Sojump platform were automatically converted into a Microsoft Excel sheet. After the data cleaning, we converted the Excel sheet into a database file for the analysis.

We used SAS (version 9.2 for Windows; SAS Institute) for the statistical analyses. The median (interquartile range (IQR)) was used to describe in continuous variables, and percentages were used to present categorical variables.

We used the χ^2^ test of categorical variables to detect the differences between different populations in each IYCF indicators. *P*-values less than 0.05 were considered statistically significant.

### Ethical approval

The study was approved by the Ethical Committee of the Capital Institute of Pediatrics in Beijing. Each WeChat questionnaire contained an electronic informed consent which the participating caregivers read and clicked “Agree to participate” before they answered the questions.

## RESULTS

### Response rate

There were 1274 eligible caregivers of children aged 6-23 months in Menyuan County and 1748 eligible caregivers in Datong County. **Table 1** shows that 1236 caregivers in Menyuan County and 1733 caregivers in Datong County responded by either filling in questionnaires by themselves or with the help of village doctors. The average total response rate was 98.2% in the two counties; 97.0% in Menyuan County and 99.1% in Datong County. Most (77.6%) questionnaires were filled in by caregivers, but 20.6% were filled in by village doctors who acted as interviewers; 10.0% in Menyuan County and 28.2% in Datong County. The questionnaire was filled in twice by 209 (6.9%) caregivers. Reasons for not responding included: refusal (n = 5); out of contact with caregivers (n = 22); and other reasons, such as the caregiver was illiterate (n = 14); caregiver's mobile phone was not a smartphone (n = 1); the child was in the hospital (n = 2); the child was adopted by others (n = 2); the caregiver said he/she had filled in the questionnaire, but the response could not be found on the Sojump platform (n = 7).

**Table 1:** Survey response rate in the two counties

	Menyuan County (N = 1274), % (n)	Datong County (N = 1748), % (n)	Total (N = 3022), % (n)
**Response**	97.0 (1236)	99.1 (1733)	98.2 (2969)
**Filled in by**			
Caregivers	87.0 (1108)	70.9 (1239)	77.6 (2347)
Village doctors as interviewers	10.0 (128)	28.2 (494)	20.6 (622)
**Multiple response**			
Once	97.0 (1236)	99.1 (1733)	98.2 (2969)
Twice	9.7 (123)	4.9 (86)	6.9 (209)
**No response**	3.0 (38)	0.9 (15)	1.8 (53)
Refused	0.3 (4)	0.1 (1)	0.2 (5)
Out of contact	1.1 (14)	0.5 (8)	0.7 (22)
Other reasons	1.6 (20)	0.3 (6)	0.9 (26)

**Table 2** shows the characteristics of children and their caregivers who responded in both counties; 52.4% of children were boys. Nearly 60% of responses were given by mothers, followed by fathers (27.2%) and grandparents (12%). More than 70% of respondents attended junior high school or above. The main occupations of the respondents were housework, farmer, and migrant worker.

**Table 2:** Characteristics of children and caregivers who responded in two counties

	Menyuan County (N = 1236), % (n)	Datong County (N = 1733), % (n)	Total (N = 2969), % (n)
**Children**			
**Gender**			
Boy	51.1 (632)	53.4 (925)	52.4 (1557)
Girl	48.9 (604)	46.6 (808)	47.6 (1412)
**Age in months**			
6-11	26.5 (327)	30.6 (530)	28.9 (857)
12-17	39.2 (485)	35.0 (607)	36.8 (1092)
18-23	34.3 (424)	34.4 (596)	34.3 (1020)
**Respondent**			
**Relation to the child**			
Mother	55.7 (689)	60.5 (1048)	58.5 (1737)
Median age in years (Q1, Q3)	27 (24, 30)	26 (24, 30)	27 (24, 30)
Father	26.8 (331)	27.4 (476)	27.2 (807)
Median age in years (Q1, Q3)	29 (27, 33)	29 (26, 32)	29 (26, 32)
Grandparents	15.0 (185)	10.0 (173)	12.0 (358)
Median age in years (Q1, Q3)	51 (48, 56)	52 (48, 55)	52 (48, 55)
Others	2.5 (31)	2.1 (36)	2.3 (67)
Median age in years (Q1, Q3)	40 (27, 47)	48.5 (30.8, 52)	41 (30, 51)
**Education**			
Primary school or below	43.4 (536)	16.9 (292)	27.9 (828)
Junior high school	42.2 (522)	61.7 (1070)	53.6 (1592)
Senior high school	10.0 (124)	14.1 (244)	12.4 (368)
College or above	4.4 (54)	7.3 (127)	6.1 (181)
**Occupation**			
Housework	22.6 (279)	36.4 (631)	30.6 (910)
Farmer	41.6 (514)	25.4 (441)	32.2 (955)
Migrant worker	26.6 (329)	25.6 (443)	26.0 (772)
Others	9.2 (114)	12.6 (218)	11.2 (332)

Q1 – first quartile, Q3 – third quartile

**Figure 2:**
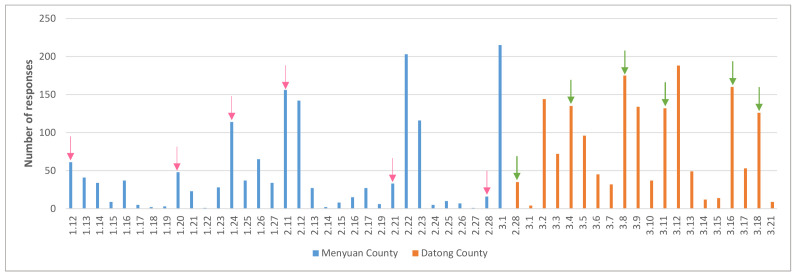
Number of responses per day in two counties. *63 responses from January 28 to February 10 were not shown, because Chinese people were celebrating the Spring Festival, during which we did not push for data collection. Days without responses were also excluded.

**Figure 2** shows the daily number of responses. The arrows in the figure indicate when we asked village doctors to remind caregivers that few caregivers responded. We found the impact of each reminder in Menyuan County usually lasted around three days. Therefore, we asked village doctors in Datong County to remind caregivers every three to four days.

**Table 3** shows a comparison of the costs, persons involved, and time spent between the previously conducted face-to-face household survey and the two WeChat surveys in this study. Using college students as interviewers, we conducted a face-to-face IYCF survey in Huzhu County, Qinghai Province in 2018. The total number of questionnaires received was 754 in Huzhu County, 1236 in Menyuan County and 1733 in Datong County. In this study in Menyuan County and Datong County, no interviewers (college students) or field supervisors were involved and no gifts were given to caregivers. The cost per questionnaire was dramatically reduced from**￥**112.7 (US$17.70) for the face-to-face survey in Huzhu County to**￥**11.8 (US$1.85) for the WeChat survey in Menyuan County and**￥**7.5(US$1.18) in Datong County. Seven days were spent in Huzhu County, 35 in Menyuan County, and 21 in Datong County.

**Table 3:** Costs, persons, and time of face-to-face and WeChat-based IYCF surveys

	Face-to-face data collection in Huzhu (￥)	WeChat-based data collection in Menyuan (￥)	WeChat-based data collection in Datong (￥)
**Costs**			
**Supervisors from Beijing**			
Travel from Beijing to county	7000	-	-
Travel from Xining to county	600	-	-
Hotel	2240	-	-
Food	800	-	-
Payment	8000	1600	1200
Insurance	200	-	-
**Interviewers (college students)**			
Travel from Xining to county	600	-	-
Hotel	11 760	-	-
Food	7800	-	-
Payment	7800	-	-
Insurance	338	-	-
**County coordinators**			
Payment	4000	800	800
**Township coordinators**			
Food	2400	-	-
Payment	3600	1200	2000
**Village doctors**			
Payment for collecting name lists	950	2100	1740
Payment for survey arrangement	950	8400	6960
**Caregivers**			
Gifts	5000	-	-
**Other cost**			
Local travel from county to villages	12 000	-	-
Stationary and printing	1430	-	-
Renting software	7540	480	240
**Total**	85 008	14 580	12 940
**Number of questionnaires received**	754	1236	1733
**Per questionnaire**	112.7	11.8	7.5
**Persons involved**			
Supervisors	2	1	1
Interviewers	26	-	-
Province coordinators	1	1	1
County coordinators	2	1	1
Township coordinators	36	12	20
Village doctors	38	105	87
**Total**	105	120	110
**Time spent (days)**	6	35	21

IYCF – infant and young child feeding

**Table 4** shows key IYCF indicators in both counties; 90.4% of children aged 6-8 months were given solid, semi-solid, or soft foods (ISSSF). Children who met MDD, MMF, and MAD accounted for 63.4%, 55.6%, and 39.3%, respectively. Children who consumed iron-rich or iron-fortified food (CIRIFF) accounted for 92.1%. Only 27.9% of children continued breastfeeding at 12-23 months (CBF).

**Table 4:** Key infant and young child feeding indicators in both counties

	Menyuan County (N = 1236)	Datong County (N = 1733)	Total (N = 2969)	Filling in once (N = 2760)	Filling in twice – 1st (N = 209)	Filling in twice – 2nd (N = 209)		
	**% (n)**	**% (n)**	**% (n)**	**% (n)**	**% (n)**	**% (n)**	P_1_*	***P_2_†***
Introduction of solid, semi-solid, or soft foods 6-8 mo‡	86.9 (106/122)	92.1 (232/252)	90.4 (338/374)	91.0 (315/346)	82.1 (23/28)	90.5 (19/21)	0.12	0.41
Minimum dietary diversity	62.6 (774)	63.9 (1107)	63.4 (1881)	64.2 (1772)	52.2 (109)	60.3 (126)	<0.001	0.09
Minimum meal frequency	57.0 (705)	54.6 (946)	55.6 (1651)	56.7 (1566)	40.7 (85)	54.6 (114)	<0.001	0.004
Minimum accepted diet	39.4 (487)	39.2 (679)	39.3 (1166)	40.3 (1111)	26.3 (55)	38.8 (81)	<0.001	0.01
Consumption of iron–rich or iron fortified foods	92.9 (1148)	91.5 (1586)	92.1 (2734)	92.5 (2552)	87.1 (182)	92.3 (193)	0.01	0.08
Continued breastfeeding at 12-23 mo§	21.2 (191/903)	33.0 (397/1202)	27.9 (588/2105)	28.1 (551/1963)	26.1 (37/142)	24.1 (35/145)	0.61	0.71

*Filling in once vs filling in twice – first time.

†Filling in twice-1st vs filling in twice – second time.

‡Denominator is the number of children aged 6-8 mo.

§Denominator is the number of children aged 12-23 mo.

209 caregivers of children filled in the questionnaires twice; their indicators were lower compared to children whose caregivers filled in the questionnaires only once, with significant difference for MDD, MMF, MAD, and CIRIFF. For children whose caregivers filled in the questionnaire twice, the indicators that were reported the second time were higher than those reported the first time, with significant differences for MMF and MAD (*P* = 0.004 and *P* = 0.01, respectively).

## DISCUSSION

### Principal findings

We conducted cross-sectional surveys in rural Qinghai province to explore the response rate of the WeChat-based data collection of IYCF information. We achieved a very high response rate of 98.2%. The cost per questionnaire was**￥**11.8 (US$1.85) in Menyuan County and**￥**7.5 (US$1.18) in Datong County, which was much lower than the cost of the face-to-face survey in Huzhu County in 2018 (**￥**112.7, US$17.70).

### Comparison with prior work

The validity and generalizability of any study is dependent on the recruitment of a sufficient number of participants [21]. The widespread use of WeChat made it into a potential survey tool in China, with studies already using WeChat to collect data [28-31]. Yusui et al. [28] conducted a cross-sectional study through WeChat on concerns about information regarding COVID-19 on the Internet, which showed a response rate of only 1.75% (10 304/590 000). Rujun et al. [29] conducted a cross-sectional study through WeChat on prevalence and associated factors of depression and anxiety among nurses during the outbreak of COVID-19 in China, which showed a response rate of 60.91% (3228/5300). Another cross-sectional WeChat survey study on factors associated with clinicians being willing to prescribe pre-exposure prophylaxis in China showed a response rate of 82.9% (777/937) [30].

In contrast with these studies, our study showed a very high response rate, achieved thanks to the village doctors on whom the study implementation relied; they collected lists with the names of children, sent the WeChat questionnaire QR codes to caregivers, and reminded non-responsive caregivers. The high response rate was achieved by both self-administered (caregivers) and interview-administered (village doctors) questionnaires. Although 77.6% of questionnaires were filled in by caregivers themselves, some caregivers were illiterate, unable to use smartphones, or unwilling to fill in questionnaires. We asked village doctors to interview these caregivers and fill in questionnaires – 20.6% of questionnaires were obtained by this method. We previously demonstrated a high consistency between self-administered and interviewer-administered data collection methods. The current study showed mixing the two methods in one survey can help improve the response rate. As the main health workers to provide health care services for rural residents, village doctors are trusted by caregivers [32]. Therefore, it is very important to engage village doctors to help conduct surveys in rural China. According to the name lists provided by village doctors, the median number of children aged 6-23 months per village was 10 in Menyuan County and 14 in Datong County. Therefore, the workloads of most village doctors were not heavy. We paid each village doctor ¥100 (US$15.71) as compensation for their time.

Our study also demonstrated that reminders are important for increasing the response rate, which is in line with previous studies [20]. As we did not have the WeChat account name of each caregiver in both counties, we asked village doctors to remind caregivers to fill in the questionnaire. At the beginning of the survey in Menyuan County, we found that very few caregivers responded after the first days, so we sent the name lists of the remaining children to the county coordinators, informing them to ask the village doctors to remind caregivers. The impact of each reminder usually lasted around three days in Menyuan County; therefore, we reminded the village doctors in Datong County every three to four days and repeated this five times until the village doctors provided caregivers’ reasons for not filling in the questionnaires.

The first step to encourage participation in any research study is to attract participants to the survey [33]. Therefore, we developed a report for caregivers, which consisted of scores for each question, a total score for all questions, inquiries about any feeding problems, and recommendations to resolve these problems. We asked village doctors to inform caregivers of the report before filling in the questionnaire so that they knew that this survey was not only to collect data but also to provide them with advice. After caregivers filled in the questionnaire, the report automatically appeared for them to check. Providing feedback so that respondents can benefit from information through participation can improve the people’s enthusiasm to take part in research [33-35]. Moreover, telling people in advance may increase the potential respondent’s trust in the researchers [20].

The cost advantages of using WeChat to administer questionnaires are more obvious in large surveys. In this WeChat-based data collection survey, the cost of each questionnaire was very low (**￥**11.8 (US$1.85) for Menyuan County and **￥**7.5 (US$1.18) for Datong County) because there were no interviewers, field supervisors, and gifts to caregivers. Although this survey took longer to complete than the 2018 face-to-face survey, its model is easy to replicate. Therefore, with the same personnel and money costs, WeChat-based data collection can achieve a wider geographical coverage.

The prevalence of the key IYCF indicators in our study was 63.4% for MDD, 55.1% for MMF, and 39.3% for MAD. These results agree with a large-scale paper-based face-to-face survey in poor rural areas of China in 2018, which showed 58.5% for MDD, 51.6% for MMF, and 35.1% for MAD [36]. Additionally, IYCF indicators for children whose caregivers filled in the questionnaire twice were lower than those for children whose caregivers filled in only once. The difference for six indicators were statistically significant, which implied that caregivers whose children’s feeding was poor were more likely to fill in the questionnaire twice. For children whose caregivers filled in the questionnaire twice, indicators of the second time were higher than those of the first time, and the difference for minimum meal frequency and minimum accepted diet were statistically significant.

These findings could indicate that providing a report after filling in questionnaires may improve the feeding practice of children, meaning this intervention could be incorporated into the data collection. A randomized controlled trial which provided feedback using the Outcome Questionnaire 45 (OQ-45) in a Swedish psychiatric outpatient population as an intervention found that the feedback group tended to improve more than the control group in the OQ-45 total score [37]. Choe et al. [38] also found that self-tracking feedback cannot only help convey data, but also affect people's attitudes and behaviors. Further studies need to be conducted to test the hypothesis.

### Strength and limitations

A strength of our study is the use of a WeChat IYCF questionnaire to collect data on infant feeding practices and caregivers’ feeding knowledge in two large-scale surveys. To our best knowledge, this is the first study to explore the response rates through WeChat in representative cross–sectional surveys in rural China. However, our study also has limitations. First, we used village doctors as interviewers to collect the data, which might have led to bias in the caregivers’ responses. Second, this study took place only in two counties in China, so the generalization of the results requires caution.

## CONCLUSIONS

This study showed that using WeChat in large-scale IYCF surveys can achieve a very high response rate at a low cost in rural China. Village doctors in rural areas played a very important role in achieving a high response rate. Providing feedback to caregivers after filling in questionnaires may improve their feeding practices. Therefore, this intervention could be incorporated into the data collection process. Further studies need to be conducted to test this hypothesis.
